# Health benefits and risks during 10 years after Roux-en-Y gastric bypass

**DOI:** 10.1007/s00464-019-07328-2

**Published:** 2020-01-28

**Authors:** M. Chahal-Kummen, O. B. K. Salte, S. Hewitt, I. K. Blom-Høgestøl, H. Risstad, J. Kristinsson, T. Mala

**Affiliations:** 1 Department of Endocrinology, Morbid Obesity and Preventive Medicine, Oslo University Hospital, Nydalen, PO Box 4950, 0424 Oslo, Norway; 2 Institute of Clinical Medicine, University of Oslo, Oslo, Norway; 3 Department of Gastrointestinal Surgery, Oslo University Hospital, Oslo, Norway

**Keywords:** Long-term outcomes, Gastric bypass, Weight loss, Cardiovascular risk factors, Gastrointestinal symptoms

## Abstract

**Background:**

Long-term evaluations 10 years after Roux-en-Y gastric bypass (RYGB) are limited. We report the development in weight and cardiovascular risk factors during 10 years after laparoscopic RYGB, with evaluation of gastrointestinal symptoms and quality of life (QoL) at 10-year follow-up.

**Methods:**

We performed a prospective longitudinal cohort study. Patients operated with laparoscopic RYGB from May 2004 to November 2006 were invited to 10-year follow-up consultations. Gastrointestinal Symptom Rating Scale (GSRS) questionnaire and two QoL questionnaires were used for analyses of gastrointestinal symptoms and QoL.

**Results:**

A total of 203 patients were operated; nine (4.4%) died during follow-up. Of 194 eligible patients, 124 (63.9%) attended 10-year follow-up consultations. Percent excess weight loss (%EWL) and percent total weight loss (%TWL) at 10 years were 53.0% and 24.1%, respectively. %EWL > 50% was seen in 53.2%. Significant weight regain (≥15%) from 2 to 10 years was seen in 63.3%. Remission rates of type 2 diabetes, dyslipidemia, and hypertension were 56.8%, 46.0%, and 41.4%, respectively. Abdominal operations beyond 30 days after RYGB were reported in 33.9%. Internal hernia and ileus (13.7%) and gallstone-related disease (9.7%) were the most common causes. Vitamin D deficiency (<50nmol/L) was seen in 33.3%. At 10 years, bothersome abdominal pain and indigestion symptoms (GSRS scores ≥3) were reported in 42.9% and 54.0%, respectively, and were associated with low QoL.

**Conclusion:**

We observed significant weight loss and remission of comorbidities 10 years after RYGB. Significant weight regain occurred in a substantial subset of patients. Gastrointestinal symptoms were common and negatively impacted QoL.

Roux-en-Y gastric bypass (RYGB) surgery is commonly applied for the treatment of morbid obesity. Short-term and medium-term outcomes are well documented and include health benefits such as significant weight loss, improvement in obesity-related comorbidities and improved quality of life (QoL) [1–5]. A limited number of studies reports on outcomes 10 years and beyond after RYGB [6–18]. Available studies indicate sustained weight loss and high remission rates of diabetes, hypertension, and dyslipidemia after RYGB. The follow-up rates, however, are often low.

Long-term evaluations on gastrointestinal symptoms after RYGB are limited. We have previously observed high rates of such symptoms 2 and 5 years after RYGB [19, 20]. Long-term evaluations and knowledge of such symptoms may improve the quality of health care for these patients.

Pending results from national and multinational databases will enlighten long-term outcomes after bariatric surgery. However, prospective cohort studies may supplement larger register-based studies, with in-depth individual evaluations. A single-center cohort study aiming to report weight development and remission of metabolic comorbidities, gastrointestinal symptoms and QoL 10 years after RYGB, were performed. We have previously reported 5-year follow-up data from this cohort [2].

## Materials and methods

### Setting

We performed a prospective longitudinal cohort study at the Department of Morbid Obesity and Bariatric Surgery, Oslo University Hospital. The institution has been a tertiary referral center for bariatric surgery since 2004, in recent years operating 250–300 patients annually. Laparoscopic RYGB was the dominating bariatric procedure performed during the inclusion period.

### Study design

Patients operated at our department during May 2004 to November 2006 were invited to 10-year follow-up. Preoperative and follow-up consultations were performed at the outpatient ward. Main aims were development of weight and cardiovascular risk factors during 10 years after RYGB. Postoperative complications, nutritional deficiencies, and gastrointestinal symptoms were secondary aims.

The study was approved by the Regional Committee for Medical and Health Research Ethics (no. 2015/142), Health Region South-East, Norway. All participants provided written consent for study participation.

### Surgery

Patients were advised a three-week low-calorie diet (1000 kcal/day) prior to surgery. RYGB was performed laparoscopically in all patients, with a gastric pouch of about 25 ml, an antecolic alimentary limb of 150 cm, and a biliopancreatic limb of 50 cm [21]. The gastrojejunostomy was made using a 45 mm linear stapler and the defect after the stapler was closed with running sutures from each corner. Mesenteric defects were not routinely closed during the study period. Patients were operated by the same two surgeons.

### Follow-up

Patients were offered follow-up consultations at 6–8 weeks, 6 months, 1, 2, 3, and 5 years after surgery. To reduce the risk of gallstone formation in patients with the gall bladder in situ, we recommend oral ursodeoxycholic acid (Ursofalk, Dr.Falk Pharma GmbH, Germany) 250 mg twice a day for six months after RYGB. Oral multivitamins (one tablet), iron (100–200 mg), vitamin D (800 IU), and calcium (1000 mg) supplementation daily, and intramuscular vitamin B12 injections (1 mg every third month) were routinely prescribed when discharged after surgery. Serum levels were evaluated at follow-up and recommendations for any adjustments were given as needed. Ten-year follow-up consultations were performed between June 2015 and June 2018.

### Definitions of weight loss

Percent excess weight loss (%EWL) was defined as: ((initial weight − follow-up weight)/(initial weight − ideal weight) * 100. Ideal weight corresponds to a BMI of 25 kg/m^2^. Percent total weight loss (%TWL) was defined as: ((initial weight − follow-up weight)/initial weight)) * 100. Percent weight regain (%WR) was calculated as: (current weight − weight at two years)/(preoperative weight − weight at two years) * 100, and was regarded significant if ≥ 15% [22, 23].

### Cardiovascular risk factors

Type 2 diabetes was defined as fasting plasma glucose ≥ 7 mmol/L, glycated hemoglobin (HbA1c) ≥ 6.5% or the use of diabetic medication. Hypertension was defined by systolic blood pressure ≥ 140/90 mmHg or the use of antihypertensive medication. Dyslipidemia was defined by fasting LDL cholesterol ≥ 3.0 mmol/l, HDL cholesterol < 1.0 mmol/L (men) or < 1.3 mmol/L (women), triglycerides > 1.7 mmol/l, total cholesterol/HDL cholesterol ratio > 5 or the use of lipid-lowering medication. Metabolic syndrome was defined according to the International Diabetes Federation, and included waist circumference, type 2 diabetes, hypertension, and dyslipidemia.

### Nutritional deficiencies

According to WHO’s classification, anemia was defined as hemoglobin < 12 g/dL in women and < 13 g/dL in men. Iron deficiency was defined as ferritin < 15 µg/L. Vitamin B12 and vitamin B9 deficiencies were defined as < 150 ρmol/L and < 10 nmol/L, respectively.

Levels of 25-OH-vitamin D < 50 nmol/L defined vitamin D deficiency.

### Abdominal surgery after RYGB

All abdominal reoperations during the 10 years after RYGB were registered. Reoperations were defined as early (within 30 days after surgery) and late (beyond 30 days after surgery).

### Gastrointestinal symptoms

The Gastrointestinal Symptom Rating Scale (GSRS, 1-week recall) was distributed at the 10-year follow-up consultations. GSRS yields total score and five syndrome scores (abdominal pain-, gastrointestinal reflux-, diarrhea-, indigestion-, and constipation syndrome) on a scale from 1 to 7 (1 = no discomfort, 7 = severe discomfort) [24]. We used the mean value of ≥ 3 for each syndrome as cutoff value for defining bothersome symptoms.

### Quality of life

SF-36v2 (4-week recall) was used to evaluate QoL. It provides eight health domain scores and two summary scores (physical and mental) from 0 to 100 (0 = maximum disability, 100 = no disability). SF-36v2 was scored using Health Outcomes Scoring Software version 5.1 (Optum Inc.) [25].

Obesity-related Problems scale (OP scale) was used to measure the impact of obesity on psychosocial aspects of life and also QoL. Transformed scale scores ranged from 0 (no impairment) to 100 (maximum impairment), and scores < 40 indicated mild impairment in psychosocial functioning, scores 40–59 indicated moderate impairment and scores ≥ 60 indicate severe impairment [26].

### Statistical analyses

Continuous data were compared using Student’s *t* test. McNemar's test or Chi-Square/χ^2^ test was used for comparison of categorical variables. Continuous variables are presented as means with standard deviations (SD) or median with 25th and 75th quartile, and categorical variables as number (portion in percent, %). Statistical analyses were performed using IBM SPSS® Statistics version 25 (IBM Corp, Armonk, New York, USA).

## Results

### Participants

There was no 30-day mortality after RYGB. Nine of 203 (4.4%) patients operated died during follow-up. Among 194 patients eligible for 10-year consultations, 124 (63.9%) patients attended 10-year follow-up. Of these, 121 (97.6%) patients had also met to 5-year consultations. Median duration of follow-up was 10.8 (10.7–11.0) years. Mean age at 10 year was 50.9 (9.1) years, and 95/124 (76.6%) were females.

### Weight development

**Fig. 1:**
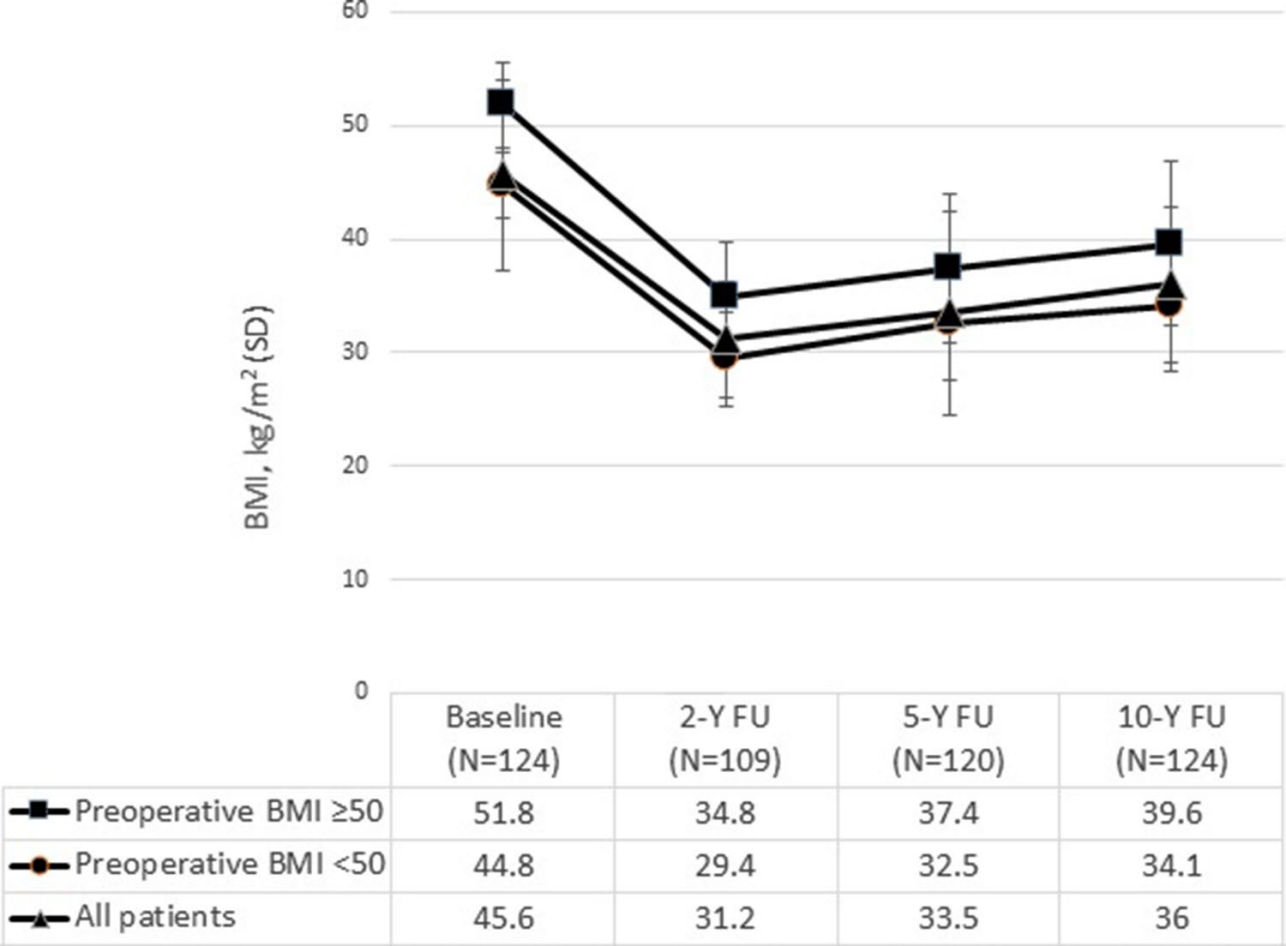
Body mass index (BMI) before and up to 10 years after laparoscopic Roux-en-Y gastric bypass (RYGB). Mean BMI (with standard deviations) during 10 years after RYGB. Significant decrease in BMI, from baseline to 10 years after RYGB, for the entire population and for patients with preoperative BMI < 50 kg/m^2^ and ≥ 50 kg/m^2^ (*P* < 0.001, paired sample *t* tests). 2-Y FU: two-year follow-up. 5-Y FU: 5-year follow-up. 10-Y FU: 10-year follow-up

Weight development is given in Table 1. At 5- and 10-year, weight loss was 37.0 (21.8) kg and 32.9 (17.3) kg, and change in BMI was 12.4 (5.7) kg/m^2^and 10.7 (6.1) kg/m^2^, respectively. In the entire cohort, %TWL > 20% was seen in 86/120 (71.7%) at 5 and in 78/124 (62.9%) at 10-year follow-up. Development in BMI during 10 years after RYGB is illustrated in Fig. 1.

**Table 1:** Weight development from baseline to 10 years after laparoscopic Roux-en-Y gastric bypass (RYGB)

	All patients			Preoperative BMI < 50 kg/m^2^ *N* = 83			Preoperative BMI ≥ 50 kg/m^2^ *N* = 41		
	Baseline	5 year	10 year	Baseline	5 year	10 year	Baseline	5 year	10 year
Weight, kg	136.0 (120.0–150.0)	97.0 (82.0–116.0)	104.8 (87.0–18.5)	129.3 (18.2)	92.5 (79.0–111.8)	99.2 (21.4)	150.0 (139.0–168.0)	109.5 (34.3)	115.5 (30.8)
BMI, kg/m^2^	45.6 (42.6–50.8)	33.5 (9.0)	36.0 (6.8)	44.8 (2.9)	32.5 (5.0)	34.1 (5.7)	51.8 (3.8)	37.4 (6.5)	39.6 (7.2)
%EWL	NA	59.5 (23.9)	53.0 (27.6)	NA	61.4 (24.4)	54.8 (28.8)	NA	52.5 (38.1)	49.4 (24.9)
%EWL > 50%	NA	61.7%	53.2%	NA	65.0%	56.6%	NA	55.0%	46.3%
%TWL	NA	26.9 (11.1)	24.1 (12.6)	NA	25.8 (10.4)	23.3 (12.5)	NA	29.1 (12.1)	25.7 (13.3)
%WR	NA	18.5 (4.8–35.7)	27.9 (5.0–49.7)	NA	18.9 (4.9–36.3)	32.1 (7.9–49.0)	NA	18.0 (2.1––32.0)	20.6 (0.0–54.8)
%WR ≥ 15%	NA	57.8%	63.3%	NA	57.5%	65.8%	NA	58.3%	58.3%

The changes during follow-up (from baseline to 5 and 10 years after RYGB, as well as from 5 to 10 years after RYGB) for the entire population and for both BMI groups, were significant for all variables (*P* < 0.05, paired sample *t* test and McNemar’s test as appropriate) except for insignificant changes in %WR ≥ 15% between 5 and 10 years after RYGB

Categorical variables are given as portion (%), continuous variables as mean (standard deviation) or median (25th and 75th quartile) as appropriate

*BMI* Body mass index, *%EWL* percent excess weight loss, *%TWL* percent total weight loss, *%WR* percent weight regain

In 83 (66.9%) patients with preoperative BMI < 50 kg/m^2^, weight loss and BMI change 5 and 10 years after RYGB was 33.6 (14.1) kg and 30.2 (16.6) kg, and 11.2 (4.8) kg/m^2^ and 9.6 (5.5) kg/m^2^, respectively. The corresponding numbers in 41 (33.1%) patients with preoperative BMI ≥ 50 kg/m^2^ was significantly higher: 44.2 (18.2) kg and 38.4 (17.7) kg, and 14.9 (6.4) kg/m^2^ and 12.8 (6.7) kg/m^2^, respectively. Patients with preoperative BMI < 50 kg/m^2^ had significantly higher weight and BMI loss (*P* < 0.005). The differences in all other parameters between the two BMI groups in Table 1 were insignificant.

### Cardiovascular risk factors

**Fig. 2:**
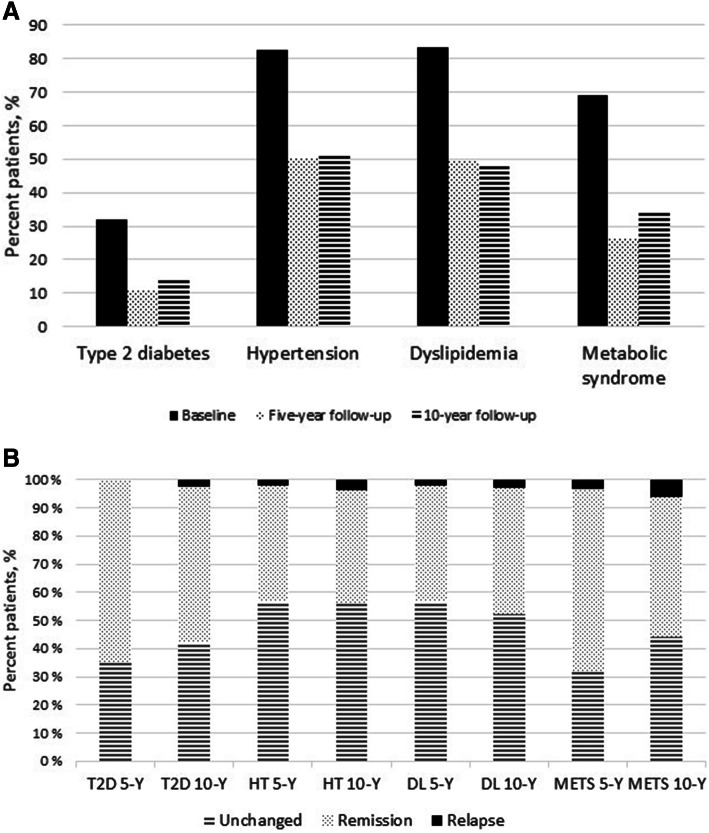
**A** Prevalence and **B** Development of cardiovascular risk factors before (*n* = 124) and 5 (*n* = 121) and 10 (*n* = 124) years after laparoscopic Roux-en-Y gastric bypass (RYGB). **A** The prevalence of comorbidities at baseline, 5-year, and 10-year follow-up: Type 2 diabetes 31.9–10.7–14.5%; hypertension 82.5–50.0–50.8%; dyslipidemia 83.3–49.6–47.6%; metabolic syndrome 69.2–26.7–34.8%, respectively. **B** The remission rates for type 2 diabetes, dyslipidemia, hypertension, and metabolic syndrome at 5 year follow-up were 64.7%, 42.3%, 42.3% and 67.2%, respectively. The corresponding remission rates at 10-year follow-up were 56.8%, 46.0%, 41.4% and 53.2%, respectively. T2D 5-Y and T2D 10-Y: type 2 diabetes at 5 and 10 years, respectively. *HT 5-Y and HT 10-Y* hypertension at 5 and 10 years, respectively, *DL 5-Y and DL 10-Y* dyslipidemia at 5 and 10 years, respectively, *METS 5-Y and METS 10-Y* metabolic syndrome at 5 and 10 years, respectively

**Fig. 3:**
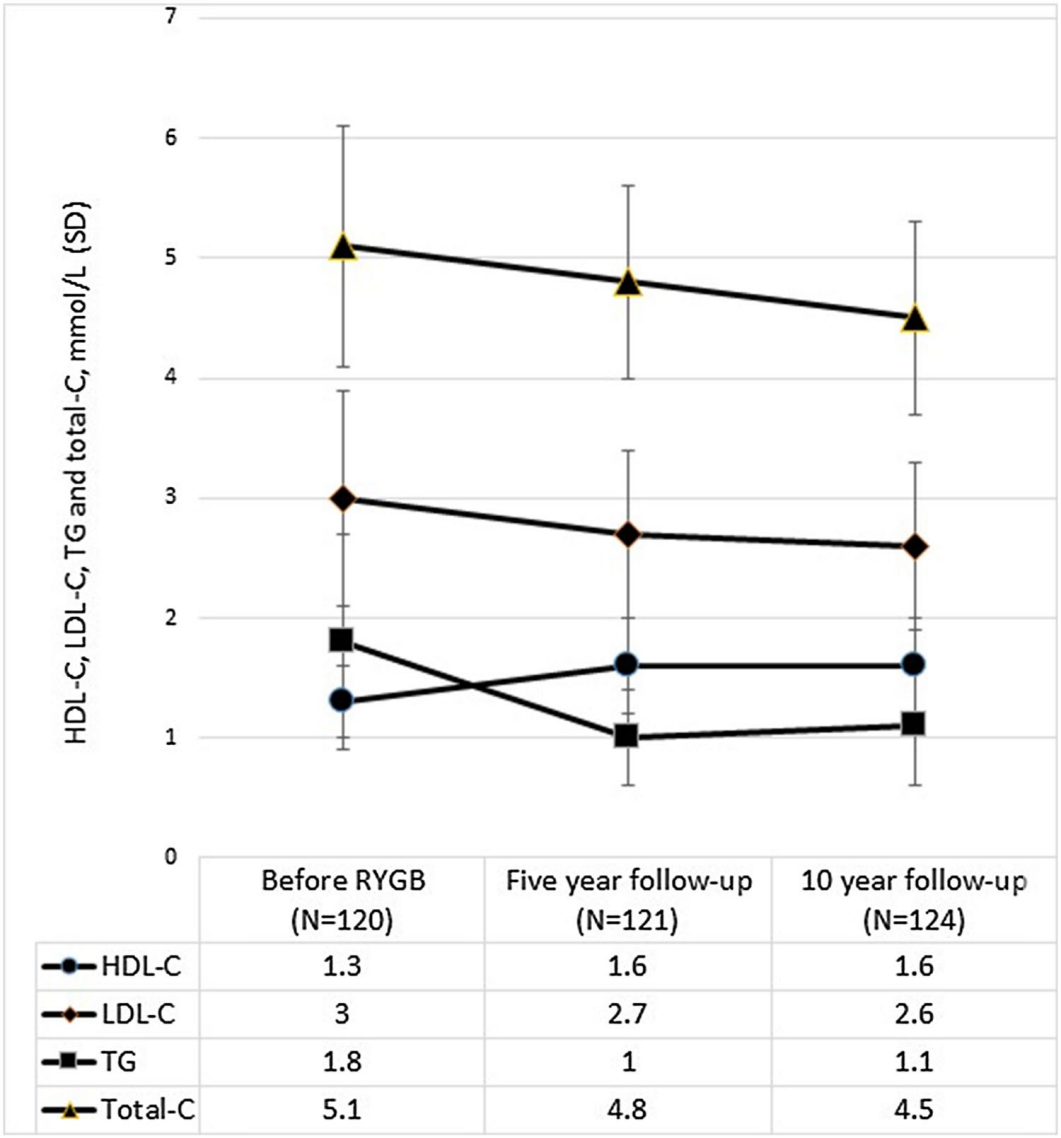
Lipid profiles during 10 years after Roux-en-Y gastric bypass (RYGB). Mean lipid values (with standard deviations). Significant increase in HDL-C, TG, and total-C and significant decrease in LDL-C from before to 5 years and 10 years after RYGB (*P* < 0.001, paired sample *t* tests). For values in mg/dl, multiply with 38.67. *HDL-C* high-density lipoprotein cholesterol, *LDL-C* low-density lipoprotein cholesterol, *TG* triglycerides, *Total-C* total cholesterol

For the entire cohort, the prevalence of type 2 diabetes, hypertension, dyslipidemia, and metabolic syndrome decreased significantly from baseline to both 5 years and 10 years after RYGB (Fig. 2). This was also true when categorized according to preoperative BMI groups of BMI < 50 kg/m^2^ and BMI ≥ 50 kg/m^2^. From 5 to 10 years after RYGB, we found a significant increase in the prevalence of metabolic syndrome for the entire population as well as for patients with preoperative BMI < 50 kg/m^2^. Lipid profiles are illustrated in Fig. 3. Median HbA1c levels were 5.8 (5.4–6.2) at baseline, and decreased significantly to 5.6 (5.4–5.9) at 5 (*P* < 0.001) and to 5.5 (5.2–5.8) at 10-year follow-up (*P* < 0.001). The decrease from 5 to 10 year follow-up was significant (*P* < 0.001).

Remission rates for comorbidities for the entire population are given in Fig. 2. 10-year remission rates for type 2 diabetes, dyslipidemia, hypertension, and metabolic syndrome for patients with preoperative BMI < 50 kg/m^2^ were: 50.0%, 44.8%, 39.1%, and 43.8%, respectively. Corresponding rates for patients with preoperative BMI ≥ 50 kg/m^2^ were: 72.7%, 48.5%, 46.7%, and 73.3%, respectively.

### Nutritional deficiencies and use of supplements

In the entire cohort, anemia was seen in 6/107 (5.6%) patients at baseline, in 19/100 (19.0%) at 5-year follow-up (*P* = 0.007) and in 25/124 (20.2%) at 10-year follow-up (*P* = 0.004). Iron deficiency was seen in 5/99 (5.1%) at baseline, in 31/102 (30.4%) at 5-year follow-up (*P* < 0.001), and in 22/124 (17.7%) at 10-year follow-up (*P* = 0.004). The reduction in iron deficiency from 5 to 10-year follow-up was significant (*P* = 0.009). During follow-up, 17/124 (13.7%) received intravenous iron infusion treatment. The prevalence of vitamin D deficiency was stable from 26/83 (31.3%) at 5 to 41/123 (33.3%) at 10 years (*P* = 0.711).

At least one vitamin supplement were used by 114/124 (91.9%) patients at 10-year follow-up. The main supplements used were vitamin B12 (85.1%), vitamin D (64.9%) and multivitamins (64.9%), calcium (49.1%), and iron (44.7%).

### Abdominal operations after RYGB

A total of 12/124 (9.7%) of patients had abdominal reoperation due to early complications. Late abdominal surgery was reported in 42/124 (33.9%) patients (Table 2).

**Table 2:** Abdominal surgery in 124 patients during 10 years after Roux-en-Y gastric bypass (RYGB)

Cause of surgery	< 30 days after RYGB	> 30 days after RYGB
Internal hernia and ileus		17 (13.7%)
Gallstone requiring cholecystectomy		12 (9.7%)
Ventral hernia/incisional hernia		8 (6.5%)
Bleeding ulcer		2 (1.6%)
Adhesiolysis		2 (1.6%)
Upper anastomotic leakage/perforation	4 (2.0%)	
Instrumental bowel perforation	3 (1.5%)	
Dilated pouch	1 (0.5%)	
Upper anastomotic stricture		1 (0.8%)
Intraabdominal abscess		1 (0.8%)
Kidney stone		1 (0.8%)
Weight regain		1 (0.8%)
Abdominal pain (diagnostic laproscopy)	1 (0.5%)	1 (0.8%)
Sepsis with subphrenic abscess	1 (0.5%)	
Intraabdominal bleeding	1 (0.5%)	
Small bowel herniation into ventral hernia	1 (0.5%)	

### Abdominal and gastrointestinal symptoms

Median GSRS syndrome scores 10 years after RYGB were abdominal pain 2.67 (1.7–3.7), reflux 1.0 (1.0–1.5), diarrhea 2.0 (1.3–3.3), constipation 1.3 (1.0–2.7), indigestion 3.0 (2.2–4.3), and total score 2.3 (1.8–3.2). Bothersome abdominal pain symptoms (scores ≥ 3) were reported by 51/119 (42.9%) patients. Bothersome symptoms for reflux syndrome, diarrhea syndrome, constipation syndrome ,and indigestion syndrome were seen in 11/119 (9.2%), 39/119 (32.8%), 25/117 (21.4%), and 65/119 (54.6%) patients, respectively. There was no significant difference in all the syndrome scores between patients with preoperative BMI < 50 kg/m^2^ and ≥ 50 kg/m^2^.

### Quality of life

**Fig. 4:**
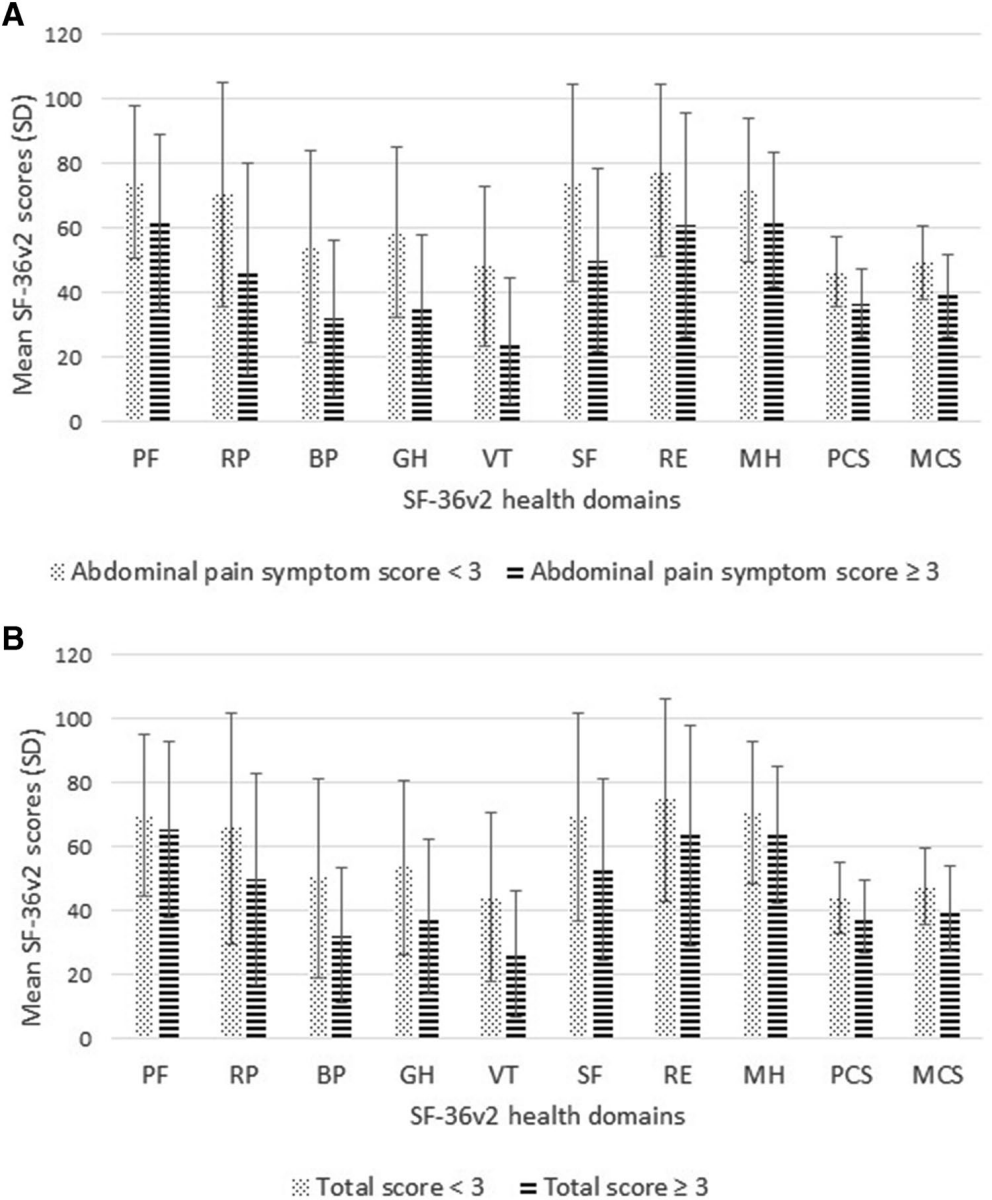
Changes in Quality of life (QoL) scores from SF-36v2 questionnaire in relation to stratified **A** abdominal pain syndrome scores and **B** total scores from the Gastrointestinal Symptoms Rating Scale (GSRS). **A** Significantly lower mean QoL scores (with standard deviations) for all health domains, in patients with GSRS abdominal pain syndrome scores ≥ 3 (*P* = 0.007 for PF, *P* < 0.001 for RP, BP, GH, VT, SF, PCS ,and MCS, *P* = 0.006 for RE ,and *P* = 0.022 for MH, independent sample *t* test). *PF* physical functioning, *RP* role physical, *BP* bodily pain, *GH* general health, *VT* vitality, *SF* social functioning, *RE* role emotional, *MH* mental health, *PCS* physical component score, *MCS* mental component score. **B** Significantly lower mean QoL scores (with standard deviations) for almost all health domains, in patients with GSRS total scores ≥ 3 (*P* < 0.005), except for PF (*P* = 0.364), RE (*P* = 0.085), and MH (*P* = 0.096) (independent sample *t* test). *PF* physical functioning, *RP* role physical, *BP* bodily pain, *GH* general health, *VT* vitality, *SF* social functioning, *RE* role emotional, *MH* mental health, *PCS* physical component score, *MCS* mental component score

Lowest scores (= most disability) among the eight SF-36v2 health domains were seen for Bodily pain 41.0 (22.0–62.0), General health 45.0 (25.0–70.5), and Vitality 37.5 (12.5–57.8). The resulting physical summary score was 42.2 (32.1–51.8) and the mental summary score was 46.3 (36.2–55.2). SF-36v2 scores according to bothersome gastrointestinal symptoms are given in Fig. 4.

Mean OP-score was 37.5 (4.2–55.2). Mild, moderate, and severe impairment of social activities were seen in 50/86 (58.1%), 15/86 (17.4%), and 20/86 (23.3%), respectively. There were no significant differences in SF-36v2 scores and OP-scores in patients with preoperative BMI < 50 kg/m^2^ and ≥ 50 kg/m^2^.

## Discussion

Our study enlightens long-term outcomes after laparoscopic RYGB. Weight loss was significant and sustained, as were beneficial effects concerning cardiovascular risk factors. However, these health benefits were accompanied by health risks such as repeated abdominal surgery, weight regain, and long-term gastrointestinal symptoms that may negatively influence QoL.

The %EWL and %TWL in our population 10 years after RYGB were comparable to what reported by others [7, 10–15, 17]. In our study, %EWL significantly decreased while %WR significantly increased from 5 to 10 years after RYGB. More focus on weight regain after RYGB is needed. Besides educating patients on appetite regulation, dietary choices, and physical activity, increased focus on mechanisms of weight regain may influence weight stability during follow-up [27].

At 10-year follow-up, patients with preoperative BMI ≥ 50 kg/m^2^ had significantly higher weight (kg) and BMI loss compared to patients with preoperative BMI < 50 kg/m^2^. We also found that patients with preoperative BMI < 50 kg/m^2^ had more weight regain 10 years after RYGB. Therefore, the differences in %EWL and %TWL in the two BMI groups were insignificant at 10 year, this in contrast to a previous study that found significant more %EWL in patients with preoperative BMI < 50 kg/m^2^ 10–13 years after RYGB [13].

The prevalence of type 2 diabetes, hypertension, dyslipidemia, and metabolic syndrome decreased significantly during follow-up (Fig. 2). The remission rate of 56.8% for type 2 diabetes in the entire population compares to other reports [11, 13]. Between 5- and 10-year follow-up, we found significant increase in metabolic syndrome for the entire population as well as for patients with preoperative BMI < 50 kg/m^2^. This increase reflects weight regain and increased weight circumference during follow-up in these patients. Beneficial lipid profiles, however, especially with significant increase in HDL, continued in the long-term as seen in Fig. 3. Patients with preoperative BMI ≥ 50 kg/m^2^ had higher remission rates for all comorbidities than patients with preoperative BMI < 50 kg/m^2^, both at 5- and 10-year follow-up, and their remission rates continued to improve to 10 years (data not shown). The metabolic benefits of RYGB may thus continue for a longer period after surgery in this subgroup of patients. A previous study evaluating 5-year outcome after RYGB has reported remission rates to be similar between the two BMI groups [28]. To our knowledge, none of the previous 10-year reports has categorized the prevalence and remission rates of comorbidities after RYGB according to the preoperative BMI. Further studies are therefore encouraged to explore our findings.

Multivitamins were used by 64.9% of our patients 10 years after surgery, which is much higher compared to another report [9]. Although vitamin D supplements were used by 64.9%, vitamin D deficiency was observed in 33.3% at 10 years. This is of concern in regard to bone health. Vitamin D deficiency in our population was lower than 58.3% reported by another study [12].

The data on surgical complications and reoperations reflect our initial experience with RYGB. Proficiency in gastric bypass surgery may include 100 patients or more [21]. During follow-up, at least 33.9% of our patients underwent abdominal surgery. Rates of abdominal surgery after RYGB otherwise vary between 14.6 and 45% [12–14]. The most common indications for surgery were internal hernia and ileus. Although not performed during the study period, closure of mesenterial defects is routine practice at our institution. Our 9.7% rate of cholecystectomy is lower than what is reported by others, which could be related to our recommended use of prophylactic ursodeoxycholic acid [6, 12].

According to the GSRS scores, 42.9% of the patients had bothersome abdominal pain symptoms 10 years after RYGB, with no significant difference between the two preoperative BMI groups. Although GSRS is a 4-week recall questionnaire, the number is still worrying. We do not have preoperative data, and the preoperative symptom burden may have been high. We have recently published before and after RYGB data reporting similar GSRS scores in 28.1% patients two years after surgery [19]. In another study, we found that 32.8% of the patients reported bothersome abdominal pain symptoms 5 years after RYGB [20]. Patients may consider abdominal pain as a part of the postoperative status and may underreport it. We found that 54.6% of the patients had symptoms related to indigestion. Increased focus on dietary choices and habits may aid in reducing these symptoms. Increased attention should be given to the risk of postoperative abdominal pain and symptoms, and information of this risk should be given to the patients in the preoperative settings.

Mean SF-36v2 and OP scale scores were comparable with one previous study; however, our observed rate of 23.3% patients with severe impairment of psychosocial functioning (OP scale) was lower than the 63.5% previously reported [29]. Importantly, as we have previously shown, bothersome abdominal pain and gastrointestinal symptoms may negatively influence QoL after RYGB. Together with postoperative abdominal pain and symptoms, focus on aspects of quality of life and of psychosocial functioning should be part of the follow-up consultations as well.

There are limitations to our study. Data on abdominal surgery after RYGB performed at other institutions were mostly patient reported, and such procedures may have been underreported. We did not have available preoperative data concerning QoL and gastrointestinal symptoms. The single-center design may reduce the external validity of our findings. Strengths of the study include the cohort design and the comprehensive and detailed follow-up from baseline to 10 years after RYGB, with paired data from several time points. Importantly, all patients were followed by in-office consultations at the same bariatric clinic. Comparable single-center and in-office long-term follow-up rates (> 10 years), has only been seen in few long-term studies [8, 11, 17, 18]. High attrition rates are a challenge in long-term evaluations post bariatric surgery. To improve follow-up we used dedicated physicians and repeated reminders with flexibility in regard to scheduling appointments. The patient-reported outcome measures add patient perspectives to our objective findings, and are highly relevant for health care quality evaluations.

## Conclusion

Significant and sustained weight loss and reduction in cardiovascular risk factors were observed 10 years after RYGB. Gastrointestinal symptoms were common and may influence QoL. The findings support the use of RYGB in selected patients.
